# Comparison of the radiolabeled PSMA-inhibitor ^111^In-PSMA-617 and the radiolabeled GRP-R antagonist ^111^In-RM2 in primary prostate cancer samples

**DOI:** 10.1186/s13550-019-0517-6

**Published:** 2019-06-03

**Authors:** Romain Schollhammer, Henri De Clermont Gallerande, Mokrane Yacoub, Marie-Laure Quintyn Ranty, Nicole Barthe, Delphine Vimont, Elif Hindié, Philippe Fernandez, Clément Morgat

**Affiliations:** 1 Nuclear Medicine Department, University Hospital of Bordeaux, Place Amélie Raba Léon, 33000, 33076 Bordeaux, France; 2 University of Bordeaux, INCIA, UMR5287, 33400 Talence, France; 3 CNRS, INCIA, UMR5287, 33400 Talence, France; 4 Department of Pathology, University Hospital of Bordeaux, 33076 Bordeaux, France; 5 Department of Pathology, University Hospital of Toulouse, 31000 Toulouse, France; 6 BioTis, Inserm U1026, Bordeaux, France

**Keywords:** Prostate cancer, PSMA, GRP-R

## Abstract

**Purpose:**

Prostate-specific membrane antigen (PSMA) and gastrin-releasing peptide receptor (GRP-R) are expressed in prostate cancer and can be targeted with radiolabeled inhibitors and antagonists. Their performances for the initial characterization of prostatic tumors have been barely evaluated but never compared. We aimed to gather comparative preclinical data of the role of PSMA and GRP-R targeting in prostate cancer.

**Procedures:**

We retrospectively studied 20 frozen prostatectomy samples with various metastatic risks of the D’Amico classification. Tissue samples were investigated by tissular microimaging using the radiolabeled PSMA inhibitor ^111^In-PSMA-617 and the radiolabeled GRP-R antagonist ^111^In-RM2. Bindings of the two radiopharmaceuticals were compared to histology and clinico-biological data (Gleason score, PSA values, metastatic risks).

**Results:**

Binding of ^111^In-PSMA-617 was high whatever the metastatic risk (*p* = 0.665), Gleason score (*p* = 0.555), or PSA value (*p* = 0.404) while ^111^In-RM2 exhibited a significantly higher binding in the low metastatic risk group (*p* = 0.046), in the low PSA value group (*p* = 0.001), and in samples with Gleason 6 score (*p* = 0.006).

**Conclusion:**

PSMA and GRP-R based imaging might have complementary performances for the initial characterization of prostatic tumors. Prospective clinical studies comparing the two tracers in this setting are needed.

## Introduction

Prostate cancer is the most common cancer in men and the third cause of cancer deaths [1]. It is also a multifocal disease as cancerous cells may arise from different locations within the prostatic gland. Thus, prostate cancer is a combination of different cancerous cells with their own metastatic risks. Prostate cancer classification, prognosis, and management are today based on the two major cell populations (Gleason score). Beside primary staging which includes multi parametric pelvic magnetic resonance imaging (mpMRI), thoraco-abdomino-pelvic computed tomography (CT), and bone scintigraphy, only ^18^F-Choline positron emission tomography/computed tomography (PET/CT) may be proposed to some patients with high metastatic risk but it has a low accuracy for detection of primary prostate cancer [2].

Attractive targets for a more specific and sensitive imaging of primary prostate cancer are the prostate-specific membrane antigen (PSMA) and the gastrin-releasing peptide receptor (GRP-R). They can be effectively targeted with radiolabeled inhibitors [3] and antagonists [4], respectively.

Prostate-specific membrane antigen (PSMA) is a type 2 glycoprotein expressed in secretory cells of prostatic epithelium. Several radiolabeled PSMA inhibitors have been developed for imaging (^68^Ga-PSMA-11, ^68^Ga-PSMA-617, ^68^Ga-PSMA I&T, or ^18^F-PSMA1007 [5]). Uptake of radiolabeled PSMA inhibitors correlates well with Gleason score (GS) and PSA level [6] indicating a role for this imaging procedure in high-risk prostate cancer.

The gastrin-releasing peptide receptor (GRP-R) is a G-protein-coupled receptor of the bombesin receptor family [7] which can be targeted with radiolabeled antagonists such as ^68^Ga-RM2 [8], ^68^Ga-NeoBOMB1 [9], or ^68^Ga-RM26 [10] for PET imaging. Contrarily to PSMA, GRP-R is overexpressed in low-risk prostate cancers (low Gleason score, low PSA value, and low tumor size) [11, 12]. A study of initial staging of prostate cancer on 14 patients observed a detection rate of 83%, a sensitivity of 89%, and a specificity of 81% [8].

Although few pilot clinical studies targeting PSMA or GRP-R for initial staging of prostate cancer suggest a complementary role of these imaging procedures, there have never been compared in the same patients. Therefore, in this preclinical work, we aimed to compare PSMA and GRP-R expression on the same histological samples of prostate tumors using radiolabeled probes.

## Material and method

### Patient characteristics

Twenty frozen samples of prostate cancer were available from the Department of Pathology of University Hospital of Toulouse, France. Patient samples were obtained after informed consent in accordance with the Declaration of Helsinki and stored at the “CRB Cancer des Hôpitaux de Toulouse (BB-0033-00014)” collection. According to the French law, CRB Cancer collection has been declared to the Ministry of Higher Education and Research (DC-2008-463) and obtained a transfer agreement (AC-2013-1955) after approbation by ethical committees (Conseil Scientifique du Centre de Ressources Biologiques). Clinical and biological annotations of the samples have been declared to CNIL (Comité National Informatique et Libertés). Sample characteristics’ are presented in Table 1. No patient had received neoadjuvant hormone therapy or chemotherapy. For each case, five adjacent sections were used: one for Hematoxylin-Eosin-Saffron (HES) staining and four for high-resolution microimaging (one section per radiopharmaceutical for total binding and another one for non-specific binding). An experienced pathologist manually drew tumoral areas on the HES-stained section. All patients were classified according to their metastatic risk, following the D’Amico classification [13], using clinical and biochemical criteria including tumoral size, PSA value, and Gleason score.

**Table 1:** Characteristics of the patients from which samples have been used in this study. *nd* not determined, *PSA* prostate-specific antigen

Patients	Age	Gleason score	PSA (ng/mL)	Clinical tumoral size: cT	Pathological tumoral size: pT	Metastatic risk
1	65	6 (3 + 3)	3.7	1	2c	Low risk
2	57	6 (3 + 3)	4.38	1	2	
3	51	6 (3 + 3)	3.7	2	2	
4	49	6 (3 + 3)	4.52	2	2c	
5	56	6 (3 + 3)	4.4	2	2c	
6	63	7 (3 + 4)	10	1	2c	Intermediate risk
7	66	7 (3 + 4)	10	2	2c	
8	59	7 (3 + 4)	13	2	2b	
9	67	7 (3 + 4)	12.5	1	3a	
10	67	7 (3 + 4)	14	1	3a	
11	66	7 (3 + 4)	10.4	0	3a	
12	55	7 (3 + 4)	13	1	3a	
13	56	9 (4 + 5)	26	3	3a	High risk
14	63	7 (4 + 3)	25.6	3	3b	
15	70	9 (4 + 5)	24.5	2	3b	
16	59	8 (4 + 4)	14	2	4	
17	48	7 (4 + 3)	14.28	2	3b	
18	66	7 (4 + 3)	44	2	3a	
19	53	7 (4 + 3)	20	2	3a	
20	63	7 (4 + 3)	28	2	nd	

### Radiosynthesis and quality controls of ^111^In-RM2 and ^111^In-PSMA-617

PSMA-617 and RM2 were radiolabeled with ^111^In using an automated synthesizer (GE FastLab, GE Healthcare, GEMS Benelux, Belgium). Briefly, 40 μg of RM2 (Life Molecular Imaging) or PSMA-617 (ABX GmBH) was heated at 90 °C for 5 min using microwaves or ^111^InCl_3_ (CURIUM®) and 5 mg of ascorbic acid for RM2. The raw solution was then purified on a C_18_ cartridge (WAT023501) preconditioned with 1 mL ethanol (Merck®) and 5 mL water (GE®). The final product was then eluted with 1 mL ethanol and formulated in PBS. ^111^In-RM2 and ^111^In-PSMA-617 were checked for radiochemical purity and amount using radio-UV-HPLC (Phenomenex Luna C_18_; 250 mm × 4.6 mm × 5 μm; 2.5 mL/min, *λ* = 220 nm; eluent A comprising 0.1% TFA in water, eluent B comprising acetonitrile; gradient 0–10 min, 95% to 5% A). The analytical HPLC system used was a JASCO system with ChromNAV software, a PU-2089 Plus quaternary gradient pump, a MD-2018 Plus photodiode array detector, and Raytest Gabi Star detector.

### High-resolution microimaging

#### Binding assay

Protocol edited by Reubi and co-workers for binding assays was strictly adhered [14]. Frozen samples were kept at − 80 °C. Three days before handling, samples were placed at − 20 °C. The day of the experiment, samples were pre-incubated for 10 min at 37 °C in Tris-HCl buffer at pH 7.4. A hydrophobic pen was used to surround the sample. Then, binding solution containing 10 nM (0.01–0.2 MBq) of ^111^In-RM2 (IC_50_ = 9.3 ± 3.3 nM on the GRP-R) [15] or 10 nM (0.03–0.06 MBq) of ^111^In-PSMA-617 (Kd = 5.4 ± 0.8 nM on the PSMA) [16] in Tris-HCl buffer at pH 8.2, 1% of BSA (Sigma®A2153), 40 μg/mL of bacitracin (Sigma®11,702), and 10 nM of MgCl_2_ (Sigma®M8266) was applied (ethanol content for ^111^In-RM2 was 0.003 ± 0.002% and 0.0009 ± 0.0008% for ^111^In-PSMA-617). For non-specific binding, 1 μM of ^nat^Ga-labeled RM2 or ^nat^Ga-PSMA-617 was added to determine non-specific binding (^nat^In-RM2 and ^nat^In-PSMA-617 were not available to us, we used ^nat^Ga-RM2 and ^nat^Ga-PSMA-617 which also bind with high affinity to GRP-R and PSMA, respectively). IC_50_ of Ga-RM2 for GRP-R is below 0.1 nM and K_i_ of Ga-PSMA-617 for PSMA is 6.40 ± 1.02 nM [17, 18]. Samples were incubated at 37 °C for 2 h. Afterward, samples were rinsed five times for 8 min in cold Tris-HCl buffer at pH 8.2 with 0.25% of BSA, two times for 8 min in cold Tris-HCl buffer at pH 8.2 without BSA and finally two times for 5 min in distilled water.

#### Tissular microimaging

Beta Imager-2000 (Biospace Lab) device was used to image and quantify radioactivity in the sample. Then, a Micro Imager (Biospace Lab) was used to obtain high-resolution images (radioactive and optical). Acquisition duration was about 20 h for the Beta Imager 2000 (4 × 10^6^ counts) and 15 h for the Micro Imager.

### Data analysis

Imaging analysis was performed as previously described [19].

### Statistical analysis

Data, presented as the mean ± standard deviation (SD), were compared using non-parametric *t* test (Wilcoxon test) and non-parametric one-way ANOVA (Kruskal-Wallis test). Statistical analyses were performed using GraphPad software (v 6.01, San Diego, USA). *p* values < 0.05 were considered statistically significant.

## Results

### Radiosynthesis and quality controls of ^111^In-RM2 and ^111^In-PSMA-617

^111^In-RM2 was produced with a radiolabeling yield of 78.5 ± 4.6%, radiochemical purity of 99.9 ± 0.2%, and specific activity of 1.4 ± 0.4 GBq/μmol. ^111^In-PSMA-617 was produced with a radiolabeling yield of 85.6 ± 0.2%, radiochemical purity of 100.0 ± 0.0%, and specific activity of 2.2 ± 0.5 GBq/μmol. Both radiopharmaceuticals are stable in PBS up to 4 h.

### High-resolution microimaging (HRMI)

#### Qualitative analysis

**Fig. 1:**
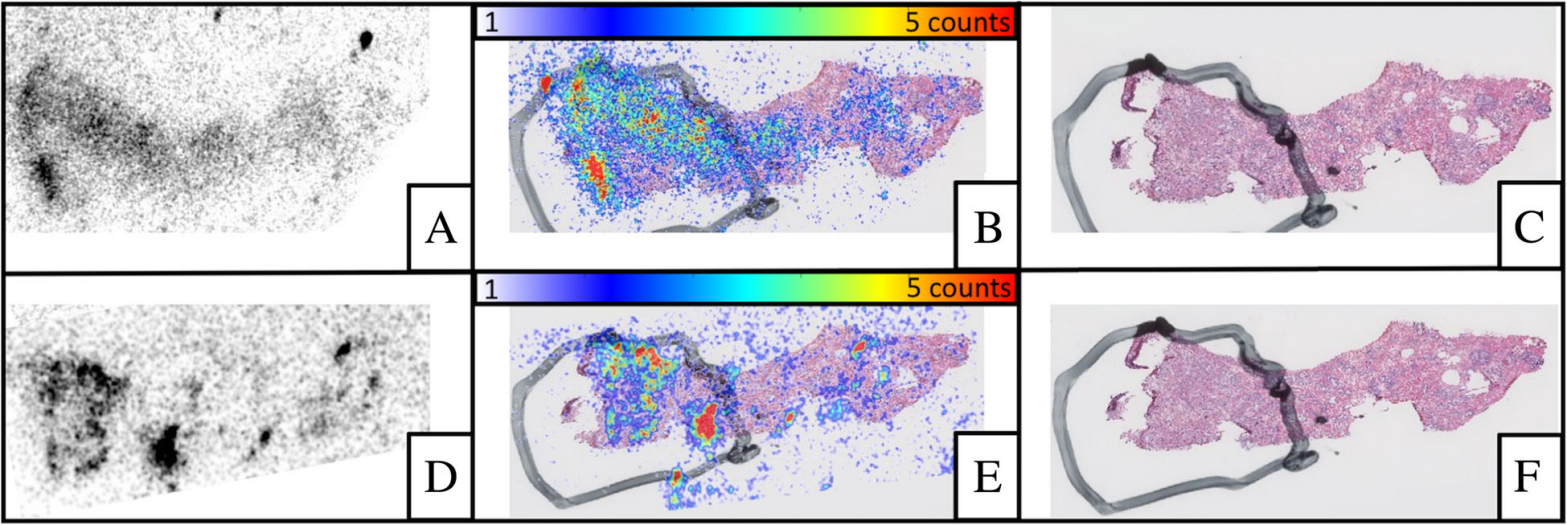
Comparison between ^111^In-RM2 (**a–c**) and ^111^In-PSMA (**d–f**) on a low-risk sample: radioactive signal (**a**, **d**), HES (**c**, **f**), and fusion images (**b**, **e**). The black line drawing corresponds to the tumoral area. There is good discrimination between tumor tissue and normal tissue on ^111^In-RM2 (tumor-to-normal ratio, TNR = 1.22) as well as on ^111^In-PSMA-617 (TNR = 2.09)

**Fig. 2:**
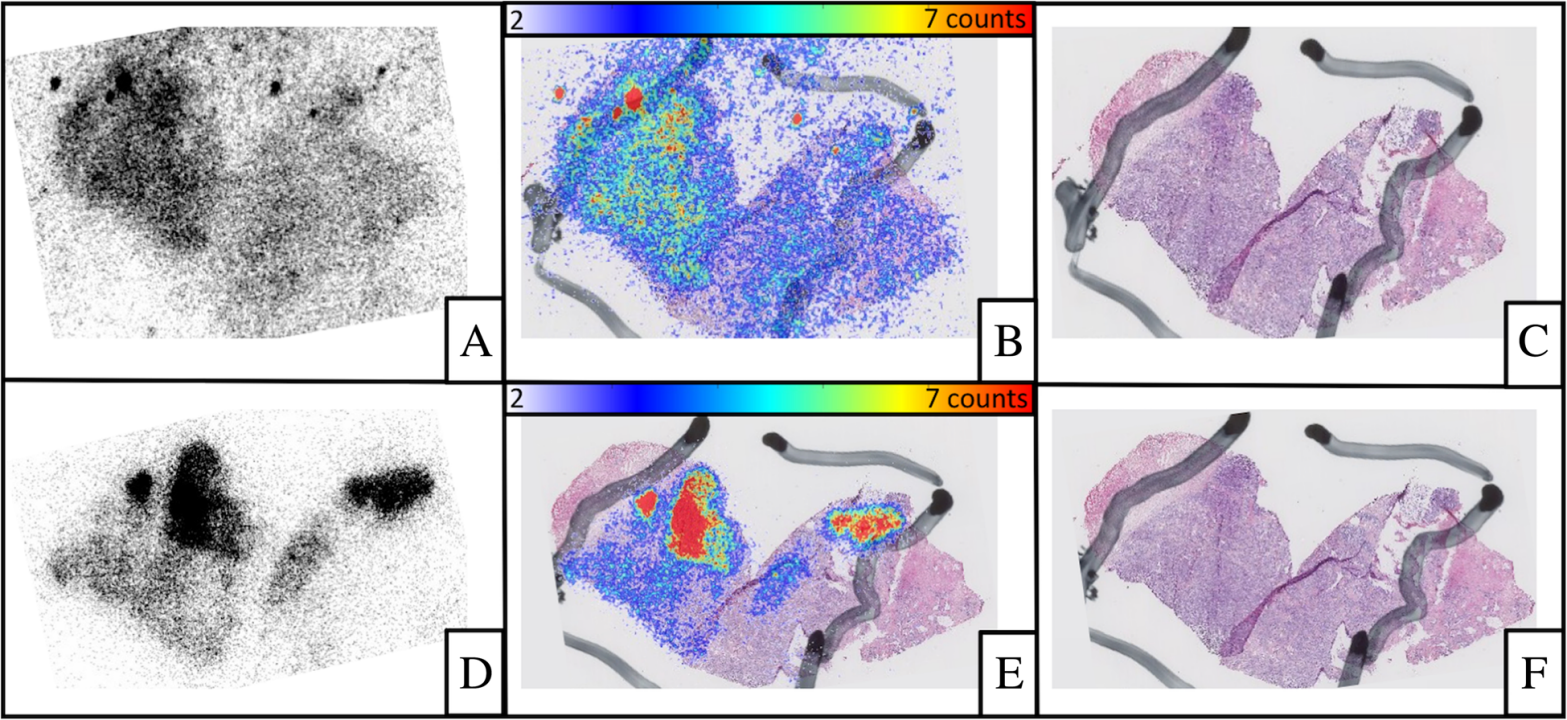
Comparison between ^111^In-RM2 (**a–c**) and ^111^In-PSMA (**d–f**) on a high-risk sample: radioactive signal (**a**, **d**), HES (**c**, **f**), and fusion images (**b**, **e**). The black line delimitation corresponds to the tumoral area. There is excellent discrimination between tumor tissue and normal tissue on ^111^In-PSMA-617 (TNR = 11.20), while the contrast is somewhat lower with ^111^In-RM2 (TNR = 1.21)

Both radiopharmaceuticals were easily detectable, without excessive noise. As shown in Fig. 1, on samples from low metastatic risk tumors, discrimination between tumoral tissues and normal tissues was good with both ^111^In-RM2 and ^111^In-PSMA-617. On high metastatic risk samples, signal-to-noise ratio was higher with ^111^In-PSMA-617 (Fig. 2).

#### Quantitative analysis

**Fig. 3:**
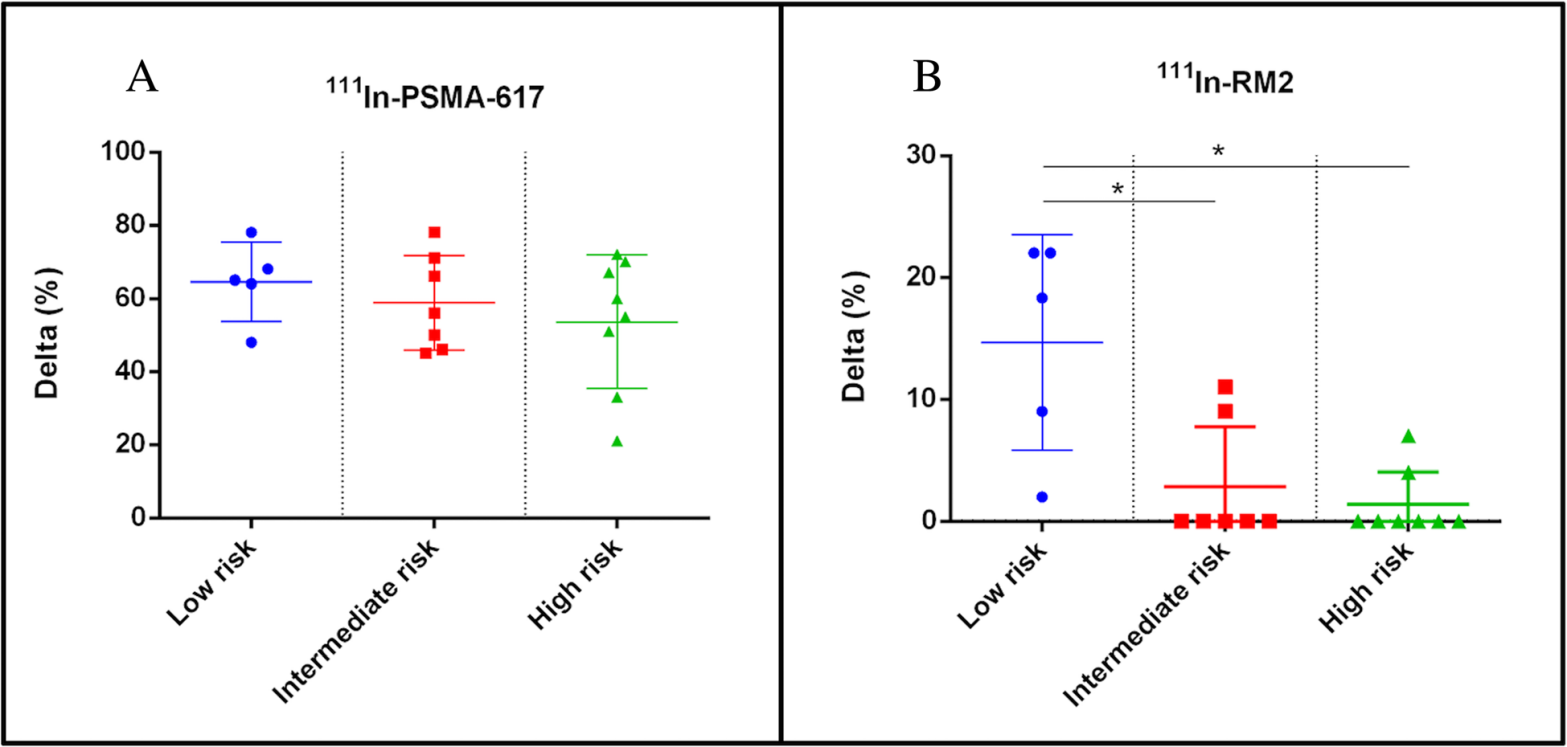
**a** ^111^In-RM2 binding in low-, intermediate-, and high-risk prostate cancer samples. ^111^In-RM2 binding is significantly higher in low metastatic risk compared to intermediate- or high-risk samples. **b** ^111^In-PSMA-617 binding in low-, intermediate-, and high-risk prostate cancer samples. Binding of ^111^In-PSMA-617 is high in all samples with no significant differences between groups. Non-parametric one-way ANOVA (Kruskal-Wallis test). *p* < 0.05 was considered significant

^111^In-RM2: The binding intensity of ^111^In-RM2 and the impact of biological, pathological, and clinical parameters are shown in Table 2. ^111^In-RM2 binding was higher in pT2 tumors compared to pT3/pT4 tumors but not significantly (9.17 ± 2.17% vs 2.82 ± 1.28%; *p* = 0.161). ^111^In-RM2 showed a significantly higher specific binding in Gleason 6 samples (Gleason 6, 14.67 ± 3.96%; Gleason 7, 2.58 ± 1.19%; Gleason 8–9, 1.33 ± 1.33%; *p* = 0.0061). ^111^In-RM2 also showed a significantly higher binding in tumors from patients with PSA < 10 ng/mL compared to patients with PSA values ≥ 10 ng/mL (14.67 ± 3.96% vs 2.07 ± 0.98; *p* = 0.0012). The differences in ^111^In-RM2 binding between low- and intermediate- or high-risk patients were also significant with higher specific binding in low metastatic group (low, 14.67 ± 3.96%; intermediate, 2.86 ± 1.86%; high, 1.38 ± 0.94%; *p* = 0.046) (Table 2 and Fig. 3).

**Table 2:** Statistical analysis of ^111^In-PSMA-617 and ^111^In-RM2 bindings according to clinico-biological parameters (pathological size, Gleason score, prostate-specific antigen (PSA) value, and metastatic risk). Non-parametric one-way ANOVA (Kruskal-Wallis test) and non-parametric *t* test (Wilcoxon test). *p* <  0.05 was considered significant

Biological parameters	*n*	^111^In-PSMA-617	^111^In-RM2	*p* value
Pathological size				
pT2	8	66.00 ± 3.65%	9.17 ± 2.17%	*0.0078*
pT3&4	11	54.82 ± 4.45%	2.82 ± 1.28%	*0.0010*
*p* value		0.105	0.161	
Gleason score				
6	5	64.60 ± 4.83%	14.67 ± 3.96%	*0.0625*
7	12	54.50 ± 4.87%	2.58 ± 1.19%	*0.0005*
8–9	3	62.33 ± 8.73%	0.0 ± 0.0%	*0.0065*
*p* value		0.5554	*0.0019*	
PSA value				
< 10 ng/mL	5	64.60 ± 4.83%	14.67 ± 3.96%	*0.0625*
≥ 10 ng/mL	15	56.07 ± 4.04%	2.07 ± 0.98%	*< 0.0001*
*p* value		0.404	*0.0012*	
Metastatic risk				
Low	5	64.60 ± 4.83%	14.67 ± 3.96%	*0.0625*
Intermediate	7	58.86 ± 4.90%	2.86 ± 1.86%	*0.0156*
High	8	53.63 ± 6.44%	1.38 ± 0.94%	*0.0078*
*p* value		0.665	*0.0046*	
Total	20	58.2 ± 14.82%	5.2 ± 7.65%	*< 0.0001*

#### ^**111**^In-PSMA-617

The binding intensity of ^111^In-PSMA-617 and the impact of biological pathological and clinical parameters are shown in Table 2. There was no significant difference in ^111^In-PSMA-617 binding intensity between groups, whether considering pT stage (pT2 vs pT3/pT4; *p* = 0.105), Gleason score (Gleason 6, 64.60 ± 4.83%; Gleason 7, 54.50 ± 4.87%; Gleason 8–9, 62.33 ± 5.04%; *p* = 0.5554), or PSA value < 10 ng/mL or ≥ 10 (64.60 ± 4.83,vs 56.07 ± 4.04%; *p* = 0.404). Again, the differences in binding between low- and intermediate- or high-risk patients were not significant (low metastatic risk, 64.60 ± 4.83%; intermediate metastatic risk, 58.86 ± 4.90%; high metastatic risk, 53.63 ± 6.44%; *p* = 0.665) (Table 2 and Fig. 3).

#### Comparison of binding intensity between ^**111**^In-PSMA-617 and ^**111**^In-RM2 according to the clinical, pathological, and biological parameters

In pT2 tumors and pT3/pT4 tumors, ^111^In-PSMA-617 binding was higher than ^111^In-RM2 (*p* = 0.0078 and *p* = 0.001, respectively). In the low PSA group, there was only a trend for higher ^111^In-PSMA-617 binding compared to ^111^In-RM2 (64.60 ± 4.83% vs 14.67 ± 3.96%, *p* = 0.0625). However, in the high PSA value group, ^111^In-PSMA-617 binding was significantly higher than ^111^In-RM2 (respectively, 56.07 ± 4.04% vs 2.07 ± 0.98%; *p* < 0.0001). There was no significant difference between the two radiopharmaceuticals in Gleason 6 score. However, in the Gleason 7 group, ^111^In-PSMA-617 was significantly higher than ^111^In-RM2 (54.50 ± 4.87% vs 2.58 ± 1.19%; *p* = 0.005). This was also the case for the few samples with Gleason 8–9 (*p* = 0.0065). ^111^In-PSMA-617 binding was significantly higher than ^111^In-RM2 binding in intermediate and high metastatic risk groups (58.86 ± 4.90% vs 2.86 ± 1.86%; *p* = 0.0156 and 53.63 ± 6.44% vs 1.38 ± 0.94%; *p* = 0.0078, respectively), while there was only a trend for higher uptake in the low-risk group (Table 2).

All results are reported in Table 2 and resumed in Fig. 3.

## Discussion

Several radiopharmaceuticals have been developed for accurate staging of prostate cancer. ^11^C-Acetate, marking lipid metabolism, cannot reliably distinguish benign prostatic hyperplasia from prostate tumors [20]. Moreover, the radiolabeled amino-acid ^18^F-FABC (^18^F-Flucicovine) did not show good diagnostic performances for characterization of primary lesions [21]. Finally, ^11^C/^18^F-Choline, also marking lipid metabolism, showed lower sensitivity than mpMRI for primary detection of prostate cancer [22]. Thus, improvements in current molecular imaging of prostate cancer appear necessary for accurate characterization of primary prostate tumors.

PSMA and GRP-R are differently overexpressed in prostate cancer, which raises hopes for molecular imaging of tumor lesions in the prostate gland. Few studies have investigated PSMA and GRP-R-based PET/CT imaging at initial staging, before radical prostatectomy. In a recent prospective study performed by Liu et al. on 50 newly diagnosed patients with prostate cancer candidates for radical prostatectomy, ^68^Ga-PSMA-617 PET/CT was able to detect 95% of primary tumors. The detection rate was similar to that of ^68^Ga-PSMA-11 PET/CT [23]. Another excellent work was performed by Touijer et al., in which authors prospectively investigate ^68^Ga-RM2 PET/CT in 16 patients before radical prostatectomy. The performances of ^68^Ga-RM2 PET/CT imaging did not significantly differ compared to mpMRI in terms of sensitivity, specificity, and accuracy. Moreover, ^68^Ga-RM2 binding did not correlate with Gleason score [24]. To date, no intra-patient comparison of PSMA and GRP-R targeting at initial staging was reported. Therefore, the objective of this work was to investigate and compare in vitro the potential role of ^111^In-PSMA-617 and ^111^In-RM2 at the initial staging of prostate cancer.

Qualitative comparison of ^111^In-PSMA-617 and ^111^In-RM2 on our primary prostate cancer samples showed good detectability of both radiopharmaceuticals which is an essential quality for contrasted images in vivo. Then, we quantitatively compared ^111^In-PSMA-617 and ^111^In-RM2. When considering all metastatic risk groups together, ^111^In-PSMA-617 binding was significantly higher than ^111^In-RM2 (*p* <  0.001). ^111^In-PSMA-617 binding was high and no differences were seen according to Gleason score or pre-operative PSA values (Table 2). This high ^111^In-PSMA-617 binding, whatever the characteristics of the sample, clearly reflects the ability of PSMA imaging to detect most prostate cancers [23] whatever their grade or risk [25]. Moreover, this high signal level may also be explained by a lower binding of ^111^In-PSMA-617 to normal tissues (*p* = 0.0161), resulting in a higher TNR for ^111^In-PSMA-617.

An interesting result of our work is that ^111^In-RM2 was able to discriminate low metastatic risk samples (*p* = 0.0046) and therefore low Gleason score samples (*p* = 0.0061) and samples with low PSA value (*p* = 0.0012). These results agree with the known high GRP-R expression in low-grade prostate cancer [11]. However, data from GRP-R immunohistochemistry and our results did not necessarily translate into parallel findings at patient imaging in pilot studies. For instance, the only two GRP-R imaging study, performed at the initial staging of prostate cancer, did not show any correlation (positive or negative) between SUV_max_ on PET/CT and Gleason scores [10, 24]. However, only 1/16 prostate cancers in the study by Touijer et al. and 2/17 in the study by Zhang et al were Gleason 6 [10, 24]. Larger clinical studies are needed to elucidate the potential offered by GRP-R targeting at the initial staging of prostate cancer. Comparison with PSMA would also be helpful.

In intermediate and high-risk samples, ^111^In-PSMA-617 binding was substantially higher than ^111^In-RM2 binding, in agreement with the known expression of GRP-R which decreases in higher Gleason scores [11]. ^111^In-PSMA-617 binding was also higher than ^111^In-RM2 binding in patients for whom pre-surgical PSA value was > 10 ng/mL. These results agree with the known efficacy of PSMA imaging of intra-prostatic tumors in newly diagnosed high-risk prostate cancer patients [6].

Our results may have future clinical value. Prostate cancer patients with low metastatic risk are today not eligible for radical treatments anymore but rather to active surveillance or focal treatments [26]. Moreover, upgrading in Gleason score between biopsies and radical prostatectomy occurs in about 30% of patients [27]. Hence, an imaging procedure capable to discriminate “true” low metastatic risks would be required to schedule focal treatments in this group of patients and not under-treat patients that would in fact be at higher risk. Results of this work indicate that GRP-R targeting is the only imaging procedure amenable to discriminate low metastatic risk from higher risks. We suggest that GRP-R-based imaging may be first proposed in low metastatic risk patients for biopsy guidance and follow-up of active surveillance. Absence or low uptake at GRP-R imaging would suggest a disease of higher risk (or no disease).

In newly diagnosed prostate cancer patients with intermediate and/or high metastatic risk, PSMA-based imaging is obviously the imaging procedure of choice to characterize intra-prostatic tumors. PSMA-based guided biopsies, staging or radiation treatment planning is being explored in prospective studies [28–30].

We have translated GRP-R and PSMA-based imaging in our center. Patient candidates for radical prostatectomy benefit from sequential ^68^Ga-PSMA-617 PET/CT and ^68^Ga-RM2 PET/CT. Preliminary results would support our in vitro data presented in this article.

Limitation of our study is obviously the limited number of samples studied. Moreover, the clinical outcome of patient for whom samples have been used in this study is not known, and we could not assess the prognostic value of GRP-R- and/or PSMA-based imaging and therefore their role in the follow-up of patients.

## Conclusion

In this work, we have compared GRP-R and PSMA expression in vitro on primary prostate cancer samples by means of ^111^In-RM2 and ^111^In-PSMA-617. Our results show that GRP-R and PSMA-based imaging may have a complimentary role to fully characterize prostate cancer disease, GRP-R being targeted in low metastatic risk patients while PSMA could be a valuable target in higher risks. Future prospective studies are warranted to confirm these data.

## Abbreviations

CNIL: Comité National Informatique et Libertés
CT: Computed tomography
GRP-R: Gastrin-releasing peptide receptor
GS: Gleason score
HES: Hematoxylin-Eosin-Saffron
HRMI: High-resolution microimaging
mpMRI: Multiparametric magnetic resonance imaging
PCa: Prostate cancer
PET/CT: Positron emission tomography/computed tomography
PSA: Prostate-specific antigen
PSMA: Prostate-specific membrane antigen
SD: Standard deviation
SUVmax: Maximum standard uptake value
